# Treatment plan quality assessment for radiotherapy of rectal cancer patients using prediction of organ-at-risk dose metrics

**DOI:** 10.1016/j.phro.2020.10.006

**Published:** 2020-10-19

**Authors:** Ana Vaniqui, Richard Canters, Femke Vaassen, Colien Hazelaar, Indra Lubken, Kirsten Kremer, Cecile Wolfs, Wouter van Elmpt

**Affiliations:** Department of Radiation Oncology (Maastro), GROW School for Oncology, Maastricht University Medical Centre+, Maastricht, the Netherlands

**Keywords:** Treatment planning QA, Prediction model, Overlap volume histogram (OVH), Knowledge based treatment planning, Dose–distance relation

## Abstract

**Background and purpose:**

Radiotherapy centers frequently lack simple tools for periodic treatment plan verification and feedback on current plan quality. It is difficult to measure treatment quality over different years or during the planning process. Here, we implemented plan quality assurance (QA) by developing a database of dose-volume histogram (DVH) metrics and a prediction model. These tools were used to assess automatically optimized treatment plans for rectal cancer patients, based on cohort analysis.

**Material and methods:**

A treatment plan QA framework was established and an overlap volume histogram based model was used to predict DVH parameters for cohorts of patients treated in 2018 and 2019 and grouped according to planning technique. A training cohort of 22 re-optimized treatment plans was used to make the prediction model. The prediction model was validated on 95 automatically generated treatment plans (automatically optimized cohort) and 93 manually optimized plans (manually optimized cohort).

**Results:**

For the manually optimized cohort, on average the prediction deviated less than 0.3 ± 1.4 Gy and −4.3 ± 5.5 Gy, for the mean doses to the bowel bag and bladder, respectively; for the automatically optimized cohort a smaller deviation was observed: −0.1 ± 1.1 Gy and −0.2 ± 2.5 Gy, respectively. The interquartile range of DVH parameters was on average smaller for the automatically optimized cohort, indicating less variation within each parameter compared to manual planning.

**Conclusion:**

An automated framework to monitor treatment quality with a DVH prediction model was successfully implemented clinically and revealed less variation in DVH parameters for automated in comparison to manually optimized plans. The framework also allowed for individual feedback and DVH estimation.

## 1 Introduction

Treatment plan quality is affected by human skills, experience and the manual (trial-and-error) aspects of radiotherapy planning, which introduces variability between patients, planners and institutions. Most centers lack methods to verify or compare the quality of their treatment plans throughout different time periods, after a change in planning technique or during the planning process. There is a lack of tools which are simple to use, provide individual feedback and strategies for quality assessment. The constant verification of treatment quality is paramount as it may drop with e.g. time, workload and the subjective aspect of technician’s experience.

There have been increasing developments in the automation and quality assurance (QA) of the treatment planning process. Automation has been shown to improve planning speed and efficiency and to reduce variability either using iterative optimization strategies or prior knowledge and experience: e.g. knowledge-based (KB) [1] or machine learning approaches [2]. However, as new plans are predicted based on previous plans, KB models are trained and validated using a selection of standard human optimized trial-and-error plans with inconsistencies across the population. KB planning drawbacks also include the risk that a subgroup of patients might receive a suboptimal plan, when being out of the KB model scope, and it might lack the planner’s interpretation of unique individual characteristics.

Therefore, it is necessary to have plan QA tools available [3]. Up-front prediction models of achievable treatment plan quality could allow for more realistic planning goals, increase planning efficiency and allow for establishing a QA system of the treatment planning process. Instead of relying solely on experience, the planner can be guided by an interval of attainable OAR doses derived from such a prediction model. Prediction models for QA have been approached by several anatomy-based methods such as the overlap volume histogram (OVH), principal component analysis and other geometric strategies [4], [5], [6], [7]. The OVH, where organ at risk (OAR) sparing is considered by its spatial overlap distribution with the planning target volume (PTV), has been applied to different treatment sites with success [8], [9], [10], [11].

For the pelvic region, different strategies have been developed for prostate cancer patients. Zhu et al. [6] proposed a machine learning based quantitative evaluation tool which estimates OAR sparing and provides reference in evaluating adaptive strategies. This tool was further used by Wang et al. to prospectively predict achievable dose-volume histograms (DVHs) for automatically generated plans. The OVH has been evaluated as an OAR dose predictor to improve treatment [12], [13], [14] and to assess automated planning software [15]. However, for rectal cancer patients a plan QA tool or an OVH model has not yet been described in the literature.

The aim of this study was to present a plan QA framework for rectal cancer patients with an anatomy-based OVH prediction model. First, the development and validation of the OVH model were outlined. The prediction model allows for feedback on plan quality during treatment planning for individual patients and periodic assessment of previously treated patients on a cohort level. Second, the prediction model was used to assess the introduction of a KB planning technique in clinical routine. Finally, the clinical framework structure was described. The framework was developed to automatically monitor planning quality, assist technicians in the planning process and create a database for further analysis. This is an application of a planning QA method, with (almost) real-time feedback to the technicians: we reported on our ongoing clinical experience.

## 2 Materials and methods

### 2.1 Patient selection and treatment planning objectives

Three hundred and seventy-one stage I-III rectal adenocarcinoma patients were treated in 2018 and 2019 at Maastro Clinic, The Netherlands. Institutional review board approval (MAASTRO-P0222) was granted for this study. A total of 196 patients treated with a prescription dose of 50 Gy in 25 fractions were included. Patients who received different treatment strategies, e.g. different fractionation or concomitant boost, were excluded, i.e. 175 patients. Patients were treated with a full bladder in supine position using two or three volumetric modulated arc therapy (Varian Rapid Arc) arcs. The gross target volume (GTV), which encompasses the primary tumor and possible lymph nodes, was expanded with 5 mm to yield the clinical target volume (CTV). The CTV was adapted considering the local anatomy and excluding overlapping OAR regions. A margin of 10 mm was added to the CTV to generate the planning target volume (PTV). The OARs comprised the bowel bag and the bladder. The clinical objectives, dose and volume constraints were summarized in supplementary material table ST1.

All 196 patients were divided into cohorts according to their treatment planning technique. The cohorts were distributed as in Supplementary material Fig. SF1. Ninety-five patients were treated after the introduction of KB software RapidPlan™ (Varian Medical Systems, Palo Alto, CA), they were referred to as automatically optimized cohort. Ninety-three patients were manually planned before the introduction of RapidPlan^TM^ according to our clinical protocol using the treatment planning system (TPS, Eclipse 15.5, Varian Medical Systems, Palo Alto, CA). This cohort was called the manually optimized cohort. Furthermore, a subset of 22 plans (not included in the other cohorts), 8 from 2018 and 14 from 2017, was revised, further optimized and re-planned, by an experienced technician and a medical physicist until they improved and reached an optimal status according to their judgment. This subset of re-plans was called the training cohort. The training cohort was used to train the OVH DVH prediction model and the manually and automatically optimized cohorts were used to validate the model. Both validation cohorts were further used to compare the manual planning technique with RapidPlan™.

### 2.2 Prediction model

**Fig. 1:**
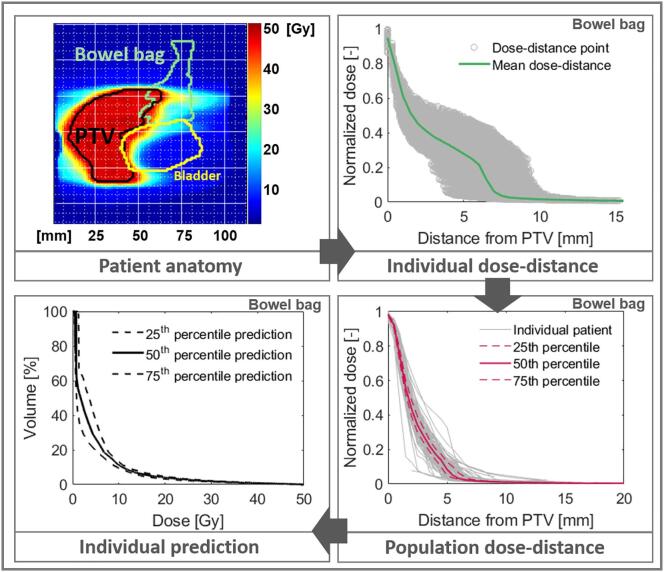
Schematic summary of the DVH prediction model. Top left: sagittal view of a patient anatomy with planned dose distribution for the PTV and OARs (bowel bag and bladder). Top right: patient-specific OAR (bowel bag) dose-distance scatter plot (in gray) and average dose-distance relation (in green). Bottom right: mean dose-distance curves for the bowel bag of all patients in the cohort (in gray) and the population’s 25^th^, 50^th^ (median) and 75^th^ percentile dose-distance curves (in pink). Bottom left: the OAR’s (bowel bag) 25^th^, 50^th^ and 75^th^ percentile DVH prediction curves for a new patient based on the population curve. (For interpretation of the references to colour in this figure legend, the reader is referred to the web version of this article.)

The DVH prediction model used in this study was based on the OVH concept: the histogram which describes the distribution of distances of an OAR with respect to the tumor [4]. It considers the dose fall off from the PTV towards each OAR; on average, as the distance from the PTV increases, the dose to the OAR decreases. This OVH DVH prediction model, which followed the method described by Petit and van Elmpt [10], was summarized and explained in Fig. 1. The first step was to derive dose-distance curves for the OARs of each patient of the training cohort. The dose-distance points, where each point shows the dose at the distance from the PTV, were displayed at the top right panel. From these points, the patient dose-distance curve was calculated, which described the relationship between the average dose in the OAR voxels at a specific distance from the PTV: steeper curves indicate a better OAR sparing. As each cohort was subject to the same treatment set-up, the dose–distance relation for each OAR was robust and almost patient independent [10]. The second step was to derive the training cohort population dose-distance curve. For each OAR, the dose-distance curves were calculated and the population relations 25^th^, 50^th^ and 75^th^ percentile were used. Thus, a bandwidth between the best 25% and 75% of plans was generated.

From the population relation, it was possible to obtain the cohort’s expected DVH and to predict DVH metrics for a new patient. For the DVH prediction, only the population curve (the expected DVH) and the distances between PTV and OARs for a new patient, i.e. their structures delineations, were necessary (Fig. 1). The prediction model was subsequently applied to the validation cohorts. Individual patient’s dose-distance relations were generated following the method described above. By comparing individual distances to the population dose-distance and associating that distance with the dose value, an estimate of the dose to each new patient was obtained. The inverse cumulative histogram of the dose at every calculation point, weighted on the new patient’s anatomy, yielded the DVH curve.

### 2.3 Generation of treatment plan database

A computational framework was established (Fig. SF2) to automatically collect treatment plan information for QA purposes. For every rectal cancer treatment, plan specific metrics e.g. irradiation technique or (achieved and predicted) treatment doses were automatically collected and stored in a database called PlanQA. The database could be accessed for prospective purposes e.g. feedback on individual patients during treatment planning, or retrospectively e.g. for cohort analysis after modifying the treatment technique.

The framework architecture was built using a pipeline from the clinical databases, where patient information is stored, towards a DICOM® mediator. The mediator moved the DICOM® treatment objects: plan, dose matrix and contours from the TPS to the Picture Archiving and Communication System (PACS) not to interfere with the clinical workflow. The PACS was connected to the PlanQA database, where, through a series of wrapped Matlab (The Mathworks, Natick, MA) scripts, treatment metrics were calculated. PlanQA was in turn connected to the clinical electronic medical record system, from which treatment protocol information was retrieved. Finally, only calculation results were stored in the PlanQA database.

For rectal cancer patients, the parameters calculated and included in the database comprehend all the DVH metrics according to the clinical goals (Table ST1), dose predictions, dose-distances and treatment plan information, such as the total number of monitor units (MU), MU/cGy, and treatment technique specifications. However, the selection of treatment plan metrics for calculation was based on a customizable configuration file thus additional parameters could be included. Calculations could be started from a website for single or multiple patients using patient and plan identifiers. The results were visualized either in an online report or using a stand-alone Matlab graphical user interface (GUI). The GUI also allowed for offline calculations from DICOM® objects.

### 2.4 Cohort analysis and prediction comparison

The validation cohorts were used to evaluate the prediction model. The predicted mean doses to the bowel bag and bladder were compared to the achieved doses as well as the V45cm^3^ for the bowel bag and the V35% for the bladder. To assess the different planning techniques, the achieved mean and maximum doses and the total number of MUs for the validation cohorts were compared using the database results. A paired *t*-test was used to indicate statistical significance (p-value < 0.05) of differences between the predicted and achieved DVH parameter values.

## 3 Results

**Fig. 2:**
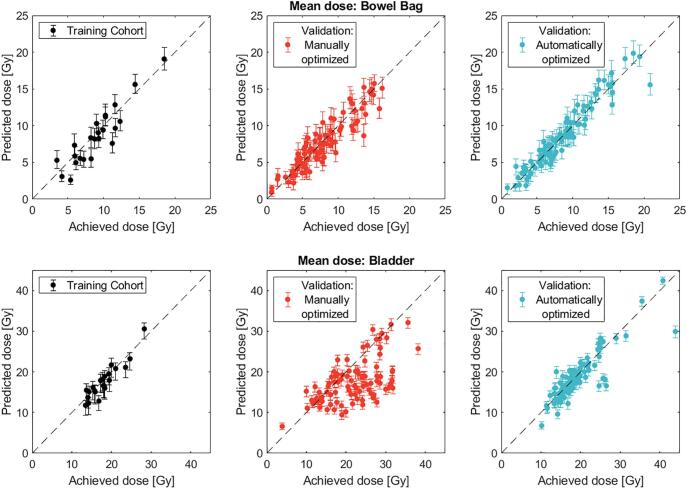
Predicted and achieved mean dose to the bowel bag and bladder for individual patients. Comparison between the training, manually and automatically optimized cohorts for the bowel bag (top) and the bladder (bottom). The predictions are derived from the median population dose–distance and the error bars correspond to the 25^th^ and 75^th^ percentiles. The diagonal line indicates where predicted and achieved results coincide.

**Fig. 3:**
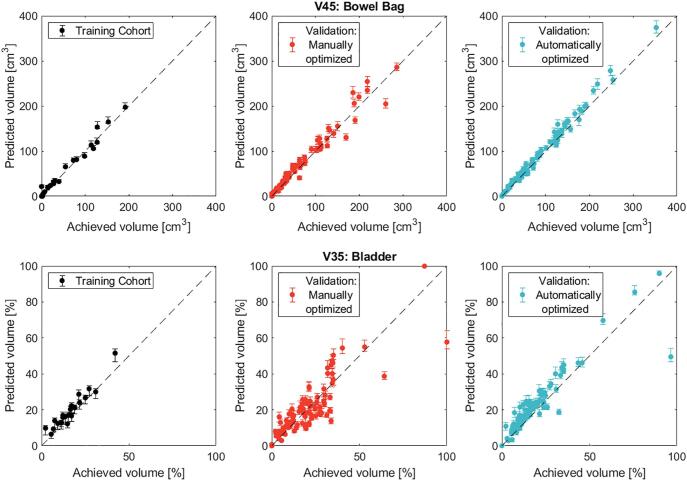
Predicted and achieved V45Gy of the bowel bag (top) and V35% of the bladder (bottom) for individual patients. Comparison between the training, manually and automatically optimized cohorts. The predictions are derived from the median population dose–distance and the error bars correspond to the 25^th^ and 75^th^ percentiles.

Variation between predicted and achieved mean doses for the automatically optimized cohort compared to the manually optimized cohort was reduced (p < 0.001) both for the mean doses to the bowel bag and the bladder (Fig. 2). This behavior was also observed for the predicted and achieved V45cm^3^ and V35% of the bowel bag and bladder, respectively (Fig. 3). The results for individual patients were summarized on the average differences between predicted and achieved doses for the treatment planning constraints (Table 1). For the manually optimized cohort, on average the prediction deviated<0.3 ± 1.4 Gy and −4.3 ± 5.5 Gy, for the mean doses to the bowel bag and bladder, respectively; for the automatically optimized cohort a smaller deviation was observed: −0.1 ± 1.1 Gy and −0.2 ± 2.5 Gy, respectively.

**Table 1:** Average differences between predicted and achieved mean dose to the bowel bag and bladder, bowel bag V45Gy and bladder V35%. The volume metrics were normalized to the respective OAR volume.

			Training cohort		Manually optimized (Validation cohort)		Automatically optimized (Validation cohort)	
			Mean ± SD	Range (min–max)	Mean ± SD	Range (min–max)	Mean ± SD	Range (min–max)
Bowel bag	ΔDmean	Absolute [Gy]	0.0 ± 1.0	−1.6–1.7	0.3 ± 1.4	−2.6–5.0	−0.1 ± 1.1	−2.6–2.7
	ΔV45	Absolute [cm^3^]	−6.3 ± 5.4	−17.5–-0.1	−0.3 ± 12.2	−43.9–55.6	−6.7 ± 11.1	−31.8–7.5
		Normalized to the bowel bag volume [%]	−0.4 ± 0.4	−1.2–0.4	−0.2 ± 0.7	−1.7–3.0	−0.4 ± 0.5	−3.0–0.8
Bladder	ΔDmean	Absolute [Gy]	−0.4 ± 2.5	−3.5–5.1	−4.3 ± 5.5	−5.3–15.7	−0.2 ± 2.5	−6.1–9.9
	ΔV35	Absolute [%]	−3.0 ± 3.6	−9.8–3.4	−0.1 ± 8.7	−15.2–42.3	−3.8 ± 3.8	−11.9–13.8
		Normalized to the bladder volume [%]	−2.9 ± 5.8	−24.2–2.3	−0.3 ± 8.9	−36.0–64.6	−2.6 ± 3.5	−6.2–15.5

**Fig. 4:**
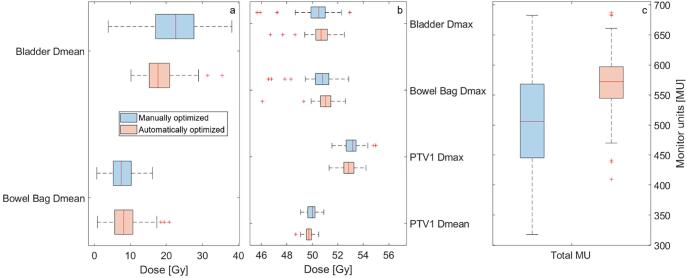
Comparison of achieved metrics from manually and automatically optimized cohorts. a) Mean bowel bag and bladder doses. b) Mean PTV and maximum PTV, bladder and bowel bag doses. c) Total number of monitor units per cohort.

**Fig. 5:**
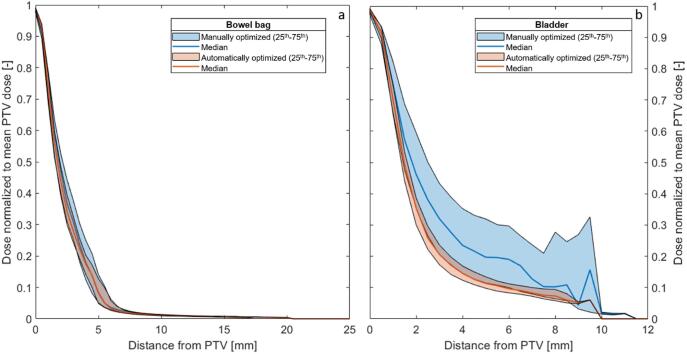
Cohort dose-distance curves for a) the bowel bag and b) the bladder for both cohorts. The area between the 25^th^ and the 75^th^ percentiles is depicted.

The mean and maximum doses to the PTV, bowel bag and bladder, were within the clinical constraints for both cohorts (Fig. 4). All volume metrics were also within the clinical constraints (not shown). The interquartile range was on average smaller for the automatically optimized cohort, indicating less variation within each parameter. For the mean and maximum dose to the bladder and PTV the interquartile range was significantly smaller (Student’s *t*-test p < 0.001) and so was for the number of MUs used in the treatment (Fig. 4c, p < 0.001), the 25^th^, 50^th^ (median) and 75^th^ percentiles were 18%, 11% and 5% higher in comparison to the manually optimized cohort; however, a slightly higher modulation, expressed in a higher MU/Gy, was seen for the automatically optimized cohort. A steeper dose fall-off for the bladder for the automatically optimized cohort was seen (Fig. 5), whereas for the bowel bag, both curves present similar behavior. This was in accordance with the mean and maximum doses (Fig. 4a) and the model premise that steeper curves indicate a better OAR sparing. The mean dose to the PTV ranged from 49.1 Gy to 50.9 Gy for the manually optimized cohort and from 48.7 Gy to 50.5 Gy for the automatically optimized cohort.

## 4 Discussion

A plan QA framework with a prediction model for DVH metrics was presented and evaluated. The framework allowed for rapid and easy access to dose and plan metrics per treatment protocol. As a first use case, the clinical introduction of a KB planning technique was evaluated. The comparison between the manually optimized and the automatically optimized cohorts showed no large dose discrepancies between both techniques, although less variation in treatment parameters using the automated technique was seen. The interquartile range was on average smaller for the automatically optimized cohort, e.g. for the total number of monitor units the range was 2.2 times higher for the manually optimized cohort. However, automatically optimized plans presented a slightly higher degree of modulation (i.e. MU/Gy).

Previous publications have also compared automated to manual treatment planning for rectal cancer patients. Wu et al. [16] reported a slightly improved homogeneity index for the PTV and significant dose reduction to the OARs. Castriconi et al. [17] implemented a KB optimization strategy for adaptive treatments which resulted in robust, better or equivalent clinical plans and a significantly lower dose to the OARs (up to 3 Gy to the bowel bag). For other sites, such as the prostate [18], [19], [20], head and neck [21] and esophagus [22], automated planning software showed superior consistency and less variation than manual planning, which demonstrates the reduction of intra- and inter-planner subjective dependency. However, to establish an acceptable model for automated planning, it is important to manually fine-tune the optimization objectives during model training [23] and to use a large variety of patients to validate the model, i.e. a cohort as heterogeneous as the one used for training is recommended [24]. Moreover, the stability of the automated planning algorithm should be evaluated over large cohorts from daily practice as it is not clear how plans are produced or if they are the best option for each patient, due to the black box aspect of commercial algorithms [15].

The prediction model from Petit and van Elmpt [10] was in this study for the first time applied to cohorts of rectal adenocarcinoma patients treated with VMAT. Based on a database of previously calculated dose-distances, this model predicted the DVH curve based exclusively on the patient’s anatomy: only the contours of the PTV and selected OARs were necessary. Moreover, the model provided confidence intervals for every calculated metric. This allowed for comparison with previously treated patients and considered the heterogeneity in the population. The method is easily implementable as it does not require complex software or expert programming skills and the trained prediction model could be distributed to other centers if the population dose-distance relationships are comparable, according to treatment technique, TPS, optimization and clinical goals. The model could also be trained at different institutions, where a training cohort of around 25 patients should yield representative predictions [10].

Prediction values were closer to the achieved results for the automatically optimized cohort in comparison to the manual cohort. This was an expected result: when during manual planning OAR dose constraints are met a plan could be considered clinically usable, but might not be optimal or Pareto optimal, i.e. any further beneficial dose redistribution to one OAR would be detrimental to other OARs. The model used to create the plans in the automatically optimized cohort was trained with a dataset of re-plans considered optimal by experts, resulting in less variation amongst the patient population, which validated the prediction model and assured the quality of the KB software. Moreover, the prediction for both dose and volume metrics was more realistic for the bowel bag in comparison to the bladder. The much larger volume of the bowel bag, different levels of bladder filling, increased distance from the PTV and different priority values set during manual plan optimization were complementary reasons for that. The dose-distance curves (Fig. 5) showed a steeper dose fall-off for the automatically optimized cohort.

Besides automating treatment planning, KB planning could be used as a QA tool: as dosimetry predictions rely on prior knowledge, e.g. dose prediction based on prior anatomical knowledge and the OVH, this data could be used for plan quality evaluation [1]. In this study, an independent DVH prediction tool for treatment plan QA was developed, trained, validated and used to assess the automated treatment planning process. Such tools are essential in the context of automated planning, as automation might decrease human awareness [15]. Plan QA models and databases can ensure treatment consistency and quality. However, a limitation of the KB approach is that model quality is a function of the training data quality: although acceptable, the quality might not be the highest and accuracy assessment becomes challenging. Wang et al. overcame this issue by creating a “ground truth” dataset of Pareto optimal plans for plan QA model performance evaluation and OVH model performance validation [25]. This sophisticated solution is likely to improve treatment quality and provides independent controls to detect systematic errors and outliers. However, their dataset was limited to prostate cancer patients and the generation of Pareto optimal plans is currently only available in a limited number of institutes.

This study shows how we developed a framework to automatically monitor planning quality, help the technicians in the planning process and create a useful database for further analysis. We suggest a concept that is flexible enough to simply store information or to add different layers of calculation on top of dicom-based treatment results, such as prediction models or e.g. outcome analysis. Radiation oncologists, medical physicists and radiotherapy technicians could use the framework for straightforward verification of historical data and improvement of plan quality. Besides assessing automated planning implementation for other treatment sites, it is possible to use this framework for cohort analysis after changes in planning techniques e.g. modification of the fractionation regimen and for assessing gradual changes in plan quality over time. Furthermore, treatment planning system operators could use the prediction model during the treatment planning process. For example, one could predict dose values for new patients who have only been contoured or whose dose calculations are finished but it is not clear if the plan could be further improved. Comparison and prediction with confidence intervals in these cases, allow for more educated clinical decisions.

We developed a clinical plan QA framework with an anatomy-based OVH prediction model and used it to assess the introduction of a KB planning technique in clinical routine. A lower degree of variation on DVH metrics was seen for automated treatment planning in comparison to manual treatment plan optimization. The framework was developed as a simple and customizable tool to automatically monitor treatment quality and provide feedback to the treatment planner.

## Declaration of Competing Interest

The authors declare that they have no known competing financial interests or personal relationships that could have appeared to influence the work reported in this paper.
